# CT evaluation of canal preparation using rotary and hand NI-TI instruments: An *in vitro* study

**DOI:** 10.4103/0972-0707.62636

**Published:** 2010

**Authors:** Shruthi Nagaraja, B V Sreenivasa Murthy

**Affiliations:** Rajiv Gandhi University of Health Sciences, Karnataka, India; 1 Department of Conservative Dentistry and Endodontics, M.S. Ramaiah Dental College, M.S. Ramaiah Nagar, MSRIT Post, Bangalore-560 054, India

**Keywords:** Canal transportation, computed tomography, nickel-titanium, ProTaper, rotary instrumentation

## Abstract

**Background:**

Controlled, uniformly tapered radicular preparation is a great challenge in endodontics. Improper preparation can lead to procedural errors like transportation of foramen, uneven dentine thickness, stripping of root canal, formation of ledge, zip, and elbow in curved canals. These procedural errors and their sequel can adversely affect the prognosis of treatment.

**Aim/Objectives:**

The present *in vitro* study aims to evaluate canal preparation based on the following factors: canal transportation, remaining dentine thickness and comparing centering ability between hand Ni-Ti K files and ProTaper rotary Ni-Ti instruments using computed tomography (CT).

**Materials and Methods:**

For evaluation, 30 mesiobuccal roots of maxillary molars were selected. Of these, 15 roots were distributed into two groups where Group 1 included hand instrumentation with Ni-Ti K-files; and Group 2 comprised ProTaper NiTi rotary system. Pre instrumentation and post instrumentation three-dimensional CT images were obtained from root cross-sections that were 1 mm thick from apex to the canal orifice; scanned images were then superimposed and compared.

**Result:**

It was observed that the manual technique using hand Ni-Ti K-file produced lesser canal transportation and maintained greater dentine thickness than the rotary ProTaper technique at middle and coronal third and this difference was statistically significant. No significant difference was seen with regard to canal transportation and remaining root dentine at apical levels. With regard to centering ratio, no significant difference was seen between both the groups at all levels.

**Conclusion:**

ProTaper should be used judiciously, especially in curved canals, as it causes higher canal transportation and thinning of root dentine at middle and coronal levels. None of the groups showed optimal centering ability.

## INTRODUCTION

The goal of biomechanical root canal preparation is to remove all the canal contents, specifically the microorganisms. This is done by enlarging and shaping the canal to allow for adequate chemical debridement, while preserving the radicular anatomy.[1] Regardless of the instrumentation technique, cleaning and shaping procedures invariably lead to dentine removal from the canal walls.[1] However, excessive dentine removal in a single direction within the canal rather than in all directions equidistantly from the main tooth axis causes what is known as ‘canal transportation’.[2]

Various studies have reported that the incidence of transportation and straightening of the canal is common with the use of stainless steel instruments.[3–7] The introduction of NiTi alloy for hand filing and later the launch of engine driven instruments have significantly altered the canal shaping procedure over past two decades. NiTi instruments possess high flexibility and torsional resistance; by using these one can effectively decrease aberrations in canals.[8]

New generation nickel-titanium rotary instruments such as ProTaper are designed to provide the minimum number of instruments that can efficiently and safely prepare a fully tapered shape.[9] A unique feature of ProTaper files is that each instrument has varying percentage tapers over the length of its cutting blades. With this geometry, rotary instruments can cut dentine more effectively and may therefore reduce torsional loads.[10] However, more aggressive cutting could produce increased canal transportation.

In the past, methods such as scanning electron microscope,[11] radiographic evaluation,[12] photographic assessment[13] and computer manipulation[7] for comparative analysis were used for assessment of canal instrumentation.

The above mentioned methods are invasive in nature, accurate repositioning of pre and post instrumented specimen is difficult, they are labor intensive, and there is a disadvantage of loss of specimen.[6] As a result the information acquired by using these methods could be misleading. With the fast growing technological advances, what is demanded are non invasive methods that would give precise information about canal preparation.

Recently, a non-destructive technology has been advocated for pre- and post instrumentation evaluation of canal. Computed tomography (CT) can render cross-sectional (cut plane) and 3D images that are highly accurate and quantifiable.[14] Comparisons using CT have provided repeatable results and also have allowed non-invasive experimentation of various aspects of endodontic instrumentation. At any level, the amount and direction of canal transportation can be viewed without loss of specimen.[14,15]

There is scanty information in literature on evaluation of canal preparation using CT. Hence the purpose of this study is to compare and evaluate canal preparation using ProTaper rotary Ni-Ti and hand Ni-Ti flex K file for canal transportation, remaining root dentine and centering ability assessed by CT.

## MATERIALS AND METHODS

30 mesiobuccal root canals of extracted maxillary first molar teeth were used. Tissue fragments and calcified debris were removed and stored in 10% formalin solution. Mesiobuccal roots with completely formed apices and angles of curvature ranging between 10° & 20°, according to the criteria described by Schneider (1971)[16] were used in the study. Second mesiobuccal canals were not included.

Access cavities were prepared using round diamond burs (Mani Inc., Tochigi-Ken, Japan).

Disto buccal and palatal roots of all teeth were separated by using a diamond disc at the furcation. To determine the working length (WL), a size 15 K-file was inserted into the remaining mesiobuccal canal until it was visible at the apical foramen. The WL of each canal was calculated to be 1 mm less than the length obtained with this initial file. Mesiobuccal roots were embedded into transparent acrylic blocks. These blocks were then placed in a dish and molten wax was poured over them for support. This created a mold for the specimen. The teeth were randomly divided into two experimental groups of 15 each.

### Scanning and imaging

**Figure 1:**
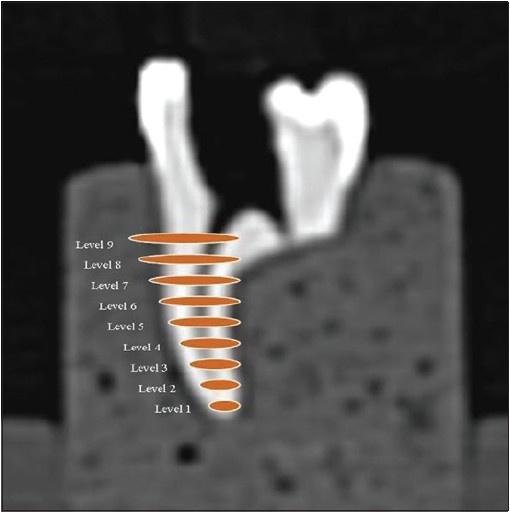
Scanning at 9 different levels

Both the groups were scanned using Spiral CT (Siemens Emotion 6 slice CT scanner) preoperatively before instrumentation. The CT scans were done using the inner ear protocol supplied by the CT scanner, at 130 KV and 130 mA, 512 × 512 pixels matrix, 1-mm-thick axial sections, 32 cm display field of view, and beam incidence at the central portion on the device used to fix the specimens. Nine levels were chosen for evaluation in the CT. Sectioning was started at 1 mm from the apex up to coronal orifice (Level 1) [Figure 1]. The images were stored in the computer's hard disk for further comparison between pre instrumentation and post instrumentation data by using DiCom software.

### Group 1 (Hand NiTi K-file)

**Figure 2:**
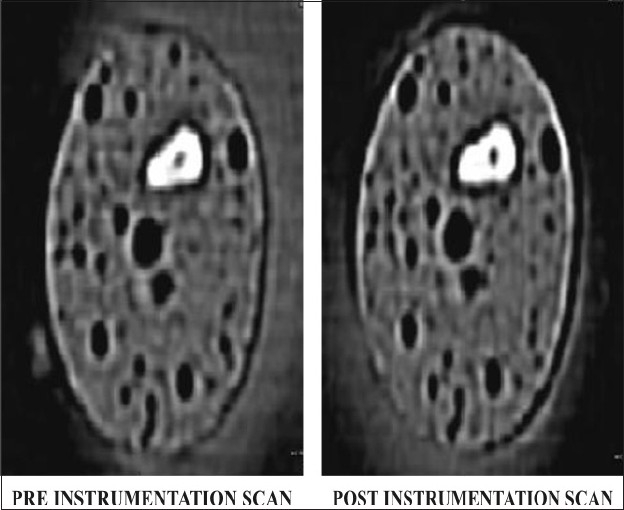
CT images of group 1

The ISO taper NiTi flex files (2% taper, Dentsply Maillefer) and Roane *et al*.[17] ‘Balanced force’ technique were used to prepare 15 root canals. Coronal preflaring was completed using size 1-3 Gates Glidden drills. The root canal was irrigated with 2 ml of 5.25% sodium hypochlorite (NaOCl) between instrumentation. RC prep (Premier) lubricant was used throughout the procedure. Each sequentially larger file was worked in a similar fashion until the apical preparation was completed with a size 30 NiTi flex file. After the size 30 NiTi flex K-file had been used to full length, the procedure was continued with a size upto 50 NiTi flex K-file keeping each file 1 mm short of WL Recapitulation with a size 30 NiTi flex K-file was carried out to avoid ledge formation. After the final irrigation had been completed, canals were dried with paper points. Each File was changed after instrumentation of five canals. Post instrumentation, teeth were then scanned under the same conditions as the initial scans [Figure 2]. Data were stored on a magnetic optical disc.

### Group 2 (ProTaper system)

**Figure 3:**
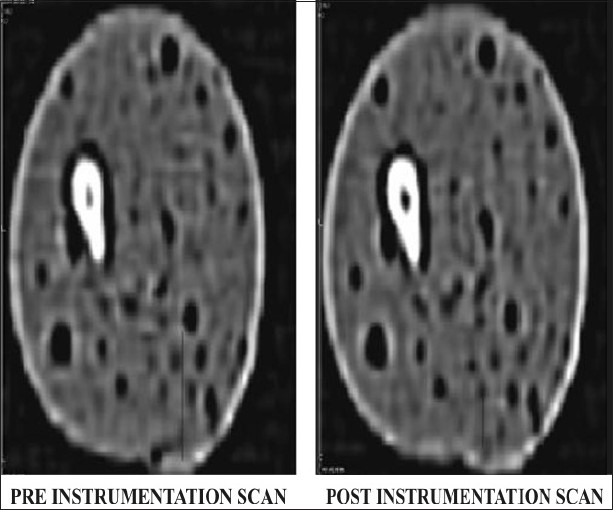
CT images of group 2

Group 2 canals were prepared using a set of ProTaper instruments (Dentsply Maillefer). Canals were prepared using torque control endodontic hand piece (X smart rotational speed 250 r.p.m.). The entire specimens were prepared according to the manufacturer's recommendation. The canals were finished when F2 reached the full WL (D1 diameter 0.25 mm). Canals were irrigated with NaOCl after each instrument, delivered by means of a gauge 27 needle, allowing for adequate back flow. RC prep (Premier) lubricant was used throughout the procedure. Post instrumentation teeth were then scanned under the same conditions as the initial scans [Figure 3]. Data were stored on a magnetic optical disc.

### Superimposition and image evaluation

**Figure 4:**
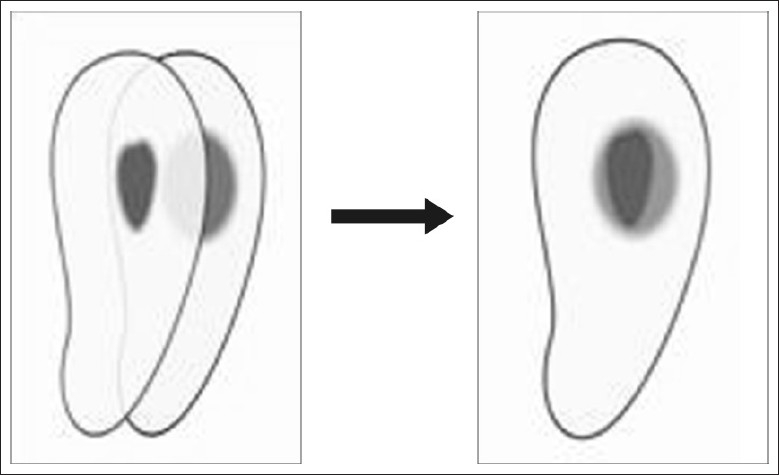
Superimposition of images

**Figure 5:**
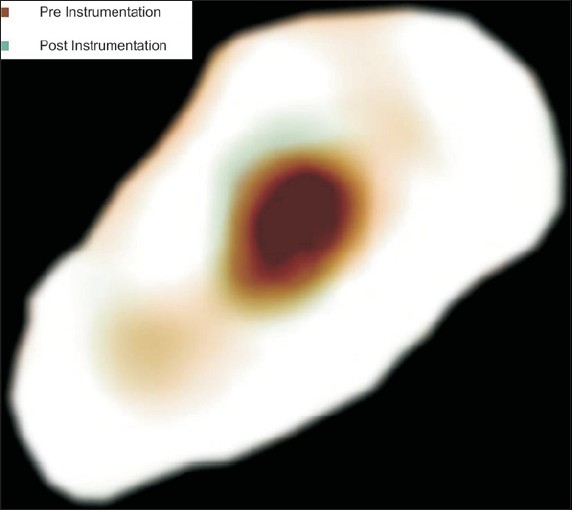
Image evaluation

Following instrumentation, the pre operative and postoperative CT reconstructions were superimposed for each group at all nine levels and the canal circumferences were traced using Adobe Photoshop software version 7.0. Narrow communications between canals were excluded. The canal centre was determined by the pixel measurement. The images were superimposed using the canal centre as reference [Figure 4 and 5].

### Evaluation of canal transportation

**Figure 6:**
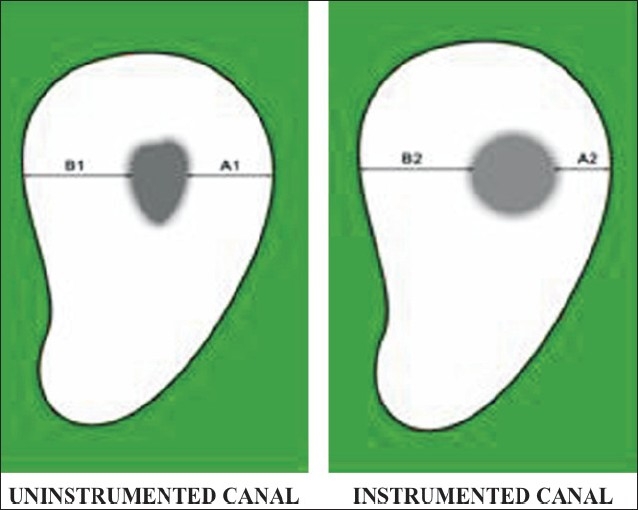
Evaluation of canal transportation

Canal transportation is defined as any undesirable deviation from natural canal paths. To compare the degree of canal transportation, a technique developed by Gambill *et al*. was used. The amount of canal transportation was determined by measuring the shortest distance from the edge of uninstrumented canal to the periphery of the root (mesial and distal) and then comparing this with the same measurements obtained from the instrumented images [Figure 6].

The following formula was used for the calculation of transportation at each level for both the groups:

{(A1-A2)-(B1-B2)}

Where A1 is the shortest distance from the mesial edge of the curved root to the mesial edge of the uninstrumented canal; B1 is the shortest distance from distal (furcation) edge of the curved root to the distal edge of the uninstrumented canal; A2 is the shortest distance from the mesial edge of the curved root to the mesial edge of the instrumented canal; and B2 is the shortest distance from distal (furcation) edge of the curved root to the distal edge of the instrumented canal.

According to this formula, a result of ‘0’ indicates no canal transportation. A result other than ‘0’ means that transportation has occurred in the canal.

### Remaining dentine thickness

**Figure 7:**
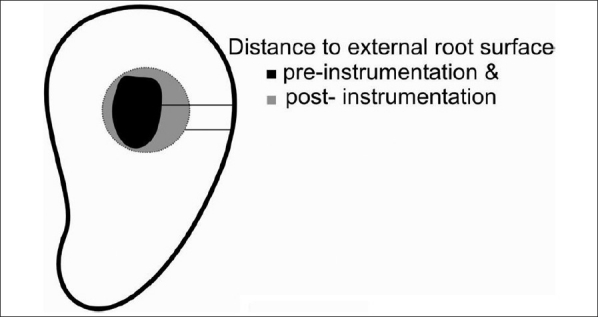
Remaining dentine thickness

For both the groups, shortest distance from the canal outline to the closest adjacent root surface was measured at each level [Figure 7].

### Evaluation of centering ability

According to Gambill *et al*.[6] ‘the mean centering ratio’ indicates the ability of the instrument to stay centered in the canal. This ratio was calculated for both the groups at each level using the following ratio:

(A1 - A2) / (B1 - B2)

Or

(B1 - B2) / A1 - A2)

If these numbers are not equal, the lower figure is considered the numerator of the ratio. According to this formula, a result of ‘1’ indicates perfect centering.

Statistical analysis used was student's t-test with 0.05 level of significance.

## RESULTS

1. Comparison of canal transportation recorded at different levels between two groups [Graph 1]

**Graph 1:**
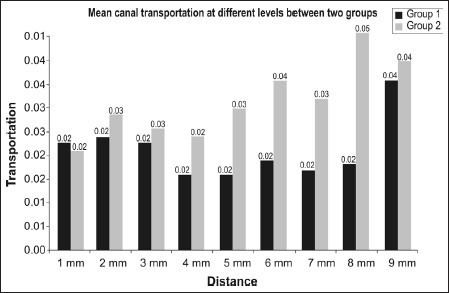
Canal transportation recorded at different levels between two groups
  There was no significant difference between the two groups with respect to transportation at 1mm, 2mm, 3mm, 4mm and 9mm (*P*>0.05). At 5mm, 6mm, 7mm and 8mm distances (*P*<0.05) greater canal transportation was noticed in Group 2 as compared to Group 1 and this difference was found to be statistically significant.
2. Comparison of remaining root dentine recorded at different levels between two groups [Graph 2]

**Graph 2:**
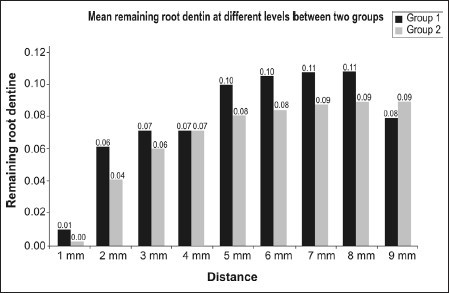
Remaining root dentine recorded at different levels between two groups
  There was no significant difference between the two groups with respect to remaining root dentine at 1 mm, 2 mm, 3 mm, 4 mm and 9 mm (*P*>0.05) however, at 5 mm, 6 mm, 7 mm and 8 mm (*P*<0.01) lesser root dentine remained in group 2 as compared to group 1.
3. Comparison of centering ratio recorded at different levels between two groups [Graph 3]

**Graph 3:**
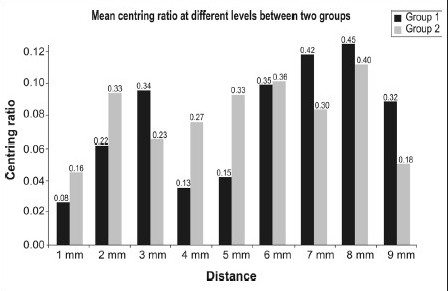
Centering ratio recorded at different levels between two groups
  There was no significant difference between the two groups with respect to centering ratio at any of the distances (*P*>0.05).

## DISCUSSION

In the present study we have evaluated the canal preparation using ProTaper rotary system and hand NiTi K-file using CT. The parameters assessed were canal transportation, centering ratio, and remaining dentine thickness.

Mesiobuccal root canals of extracted human maxillary molars were used in the present study because they usually present an accentuated curvature and mesiodistal flattening. These characteristics are additional shortcomings during the chemo mechanical instrumentation process and make cleaning and shaping of these canals more difficult, mainly in the isthmus areas. The crowns were maintained to simulate, as closely as possible, the clinical endodontic practice, in which the interference of cervical dentine projections creates tensions on the files during root canal instrumentation.[2]

The use of CT to evaluate the quality of root canal preparation has been reported to provide better results[14] than those provided by other methods like radiographic imaging,[12] cross-sectioning[6] and longitudinal cleavage.[13] In the CT imaging system, by changing the viewing parameters, it was possible to show images with more or less tooth density and detail. Once the images have been digitized, there are infinite ways in which they can be manipulated and viewed. The effective CT slices used in this study were of 1mm thickness which provided a practical and non-destructive technique for assessment of canal morphology before and after shaping.[14] In this study, all the parameters were evaluated at nine levels 1mm from the apex to straight part of the canal at equal intervals.

The first parameter evaluated was canal transportation: In this investigation, we did not see any significant occurrence of transportation at apical levels in both the groups. But there was significant transportation at middle and coronal levels when ProTaper was used.

No significant difference between the groups at the apical levels may be attributed to various factors like (a) instrumentation technique, (b) type of instrument and (c) tip design of instrument.

(a) Instrumentation technique: Balanced force technique was employed in Group 1 and Crown Down technique in Group 2 which resulted in less canal transportation in apical third. Y. Garip and M. Günday[15] indicated that shapes created with balanced force techniques are of excellent quality and are comparable to those with NiTi rotary instruments. (b) Type of instrument: NiTi files that were used in both the groups have super elasticity and shape memory which has minimized the straightening effect and hence less canal transportation.[5–8] (c) Tip design of instrument: The modified cutting tips used in both the groups reduced the undesirable changes in the curved canal unlike the conventional cutting tips.[18]

At the middle and coronal levels, Group2 (ProTaper) showed higher transportation which can be mainly attributed to progressive taper along the cutting surface in combination with the sharp cutting edges.

The second parameter evaluated was remaining root dentine. No statistical difference for remaining root dentine at apical levels between both the groups could be attributed to the non cutting modified safety tip of the ProTaper and blunt transition angle at the tip of NiTi flex K-file. But the remaining root dentine was significantly thinner at the mid-root and coronal level sections following ProTaper instrumentation. Progressively tapered design along with triangular convex cross sectional design could have led to aggressive cutting.[9,10]

The third parameter evaluated in our study was centering ability. Centering ability at all levels for both the groups was much lesser than ratio 1 and there was no significant difference between both the groups. This signifies that flexibility of Ni-Ti instruments did not positively influence the centering ratio in both the groups.[15] Other reasons which could be attributed to lower centering ability is absence of radial lands.[18] Both the groups in our study had no radial lands.

If the instrument failed to remain centered in the curved canal, it could result in irregularly shaped canals. Multiple plane preparation, both buccolingual and mesiodistal direction, is necessary when dealing with curved root canals to preserve the natural curve of flow. Transportation of the apical foramen either internally or externally, should be avoided such that the diameter at the apex is narrowest. The least remaining canal wall thickness is an indication of the safety of the instruments for preparing curved root canals. An ideal instrumentation would have equal dentine removal from the canal walls, providing successful debridement, yet it could avoid excessive thinning of root structure.[19]

The findings of the present study regarding the canal transportation produced by rotary systems in MB root canals of maxillary molars are consistent with those of Peters *et al*.,[20] who reported similar results with the ProTaper system. Other studies[15,17] found no differences among various instrumentation techniques regarding canal transportation. Glossen *et al*.[5] and Tasdemir *et al*.[21] evaluated root canal preparation with different techniques and found that rotary NiTi instruments produced less canal transportation than stainless steel or NiTi hand files. The reasons for these divergent results might be the differences in methodologies, such as tooth type, methods of assessment, instruments, and instrumentation techniques.

Comparing the instrumentation evaluated in this study from a clinical standpoint, interestingly, rotary NiTi instrumentation caused more canal transportation than conventional hand instrumentation, which is an important finding to be considered under clinical conditions. Non ISO progressive taper in ProTaper system results in more tooth cutting in middle and coronal third. However, in apical third, ISO and Non ISO taper perform equally.

The results of this study show that CT scanning is an accurate and efficient method of assessment of root canal instrumentation techniques. During instrumentation, instrument might deviate to buccal or palatal side. The bucco-palatal direction is not assessed, which could be a limitation of our study.

## CONCLUSION

According to the methodology used and on the basis of the results of this study, it can be concluded that ProTaper should be used judiciously, especially in curved canals as it causes higher canal transportation and thinning of root dentine at middle and coronal levels. This is an important finding which could be considered under clinical situations.

Research should continue to further improve instrument design, preparation techniques, and methodologies that are used to evaluate the action of endodontic instruments inside the root canal, aiming at solving the problems inherent to shaping of canals an important and difficult phase of the endodontic therapy.
